# Artificial Intelligence Technologies Supporting Nurses' Clinical Decision‐Making: A Systematic Review

**DOI:** 10.1111/jocn.70156

**Published:** 2025-11-25

**Authors:** Kristina Mikkonen, Saara Tuunainen, Anne Oikarinen, Miia Jansson, Brigitte Woo, Wentao Zhou, Wilson Tam, Anna‐Maria Tuomikoski, Pirjo Kaakinen, Jonna Juntunen, Erika Jarva

**Affiliations:** ^1^ Research Unit of Health Sciences and Technology University of Oulu Oulu Finland; ^2^ Medical Research Center Oulu Oulu University Hospital and University of Oulu Oulu Finland; ^3^ Alice Lee Centre for Nursing Studies, Yong Loo Lin School of Medicine National University of Singapore Singapore Singapore; ^4^ Oulu University Hospital The Wellbeing Services County of North Ostrobotnia Oulu Finland

**Keywords:** AI tool usability, artificial intelligence, nursing decision‐making, systematic review

## Abstract

**Background:**

The use of technology to support nurses' decision‐making is increasing in response to growing healthcare demands. AI, a global trend, holds great potential to enhance nurses' daily work if implemented systematically, paving the way for a promising future in healthcare.

**Objectives:**

To identify and describe AI technologies for nurses' clinical decision‐making in healthcare settings.

**Design:**

A systematic literature review.

**Data Sources:**

CINAHL, PubMed, Scopus, ProQuest, and Medic were searched for studies with experimental design published between 2005 and 2024.

**Review Methods:**

JBI guidelines guided the review. At least two researchers independently assessed the eligibility of the studies based on title, abstract, and full text, as well as the methodological quality of the studies. Narrative analysis of the study findings was performed.

**Results:**

Eight studies showed AI tools improved decision‐making, patient care, and staff performance. A discharge support system reduced 30‐day readmissions from 22.2% to 9.4% (*p* = 0.015); a deterioration algorithm cut time to contact senior staff (*p* = 0.040) and order tests (*p* = 0.049). Neonatal resuscitation accuracy rose to 94%–95% versus 55%–80% (*p* < 0.001); seizure assessment confidence improved (*p* = 0.01); pressure ulcer prevention (*p* = 0.002) and visual differentiation (*p* < 0.001) improved. Documentation quality increased (*p* < 0.001).

**Conclusions:**

AI integration in nursing has the potential to optimise decision‐making, improve patient care quality, and enhance workflow efficiency. Ethical considerations must address transparency, bias mitigation, data privacy, and accountability in AI‐driven decisions, ensuring patient safety and trust while supporting equitable, evidence‐based care delivery.

**Impact:**

The findings underline the transformative role of AI in addressing pressing nursing challenges such as staffing shortages, workload management, and error reduction. By supporting clinical decision‐making and workflow efficiency, AI can enhance patient safety, care quality, and nurses' capacity to focus on direct patient care. A stronger emphasis on research and implementation will help bridge usability and scalability gaps, ensuring sustainable integration of AI across diverse healthcare settings.

## What Does This Paper Contribute to the Wider Global Community?

- Highlights the critical role of AI in enhancing nursing decision‐making and patient care across diverse healthcare settings.
- Emphasises the need for user‐centred design and ethical considerations in the development and deployment of AI tools.
- Identifies gaps in research methodologies and calls for standardised, high‐quality studies to validate AI applications.
- Advocates for global adoption of AI in healthcare, including in low‐resource settings, to address disparities and improve equity in care delivery.

## 1 Introduction

At present, healthcare systems globally are facing challenges to meet the unmet needs of healthcare. Increased demand combined with scarce resources is a challenging equation (Kingston et al. 2018). Improving the quality of care without increasing resources requires innovations (Apell and Eriksson 2023; Bergman et al. 2015). Artificial intelligence (AI) could potentially be a powerful technology to accelerate innovation and support evidence‐based clinical decision‐making in healthcare. As healthcare becomes increasingly digital and data‐rich, advances in computing power and AI techniques create vast opportunities (Jiang et al. 2017; Shang 2021). In this context, generalised AI tools are designed for broad clinical applications across multiple conditions and care settings, whereas specialised AI tools target specific tasks or outcomes, such as predicting hospital readmissions or supporting neonatal resuscitation. Clarifying this distinction is important for understanding the scope and applicability of different AI solutions in nursing practice.

AI refers to computational systems designed to perform tasks traditionally requiring human intelligence, such as reasoning, adaptation, natural language processing, deep learning, and sensory interpretation (Apell and Eriksson 2023). AI‐driven technologies can augment or, in some cases, replace human interpretation and decision‐making skills (Secinaro et al. 2021). Driven by relevant clinical questions, powerful AI technologies can unlock clinically relevant information hidden among massive amounts of data that can, in turn, guide clinical decision‐making. In nursing contexts, AI may process complex patient data to detect risks, identify care priorities, and support timely interventions. The technologies also have the potential to discover new treatments (Jiang et al. 2017; Secinaro et al. 2021). Among other things, AI can help improve the accuracy of clinical decisions, care efficiency, and access to healthcare services (Gennatas and Chen 2021).

Although most healthcare professionals are nurses, the use of AI in nursing has been slow to take off (Shang 2021). According to statistics, 8%–16% of nurses' working time is spent on tasks that could be delegated to others. AI could reallocate this time to direct patient care requiring human judgement, empathy, and complex decision‐making (Robert 2019).

Many of the challenges of using AI are technical and concern improving current healthcare practices (Montemayor et al. 2022). However, ethical and governance considerations are also central, including data privacy, bias mitigation, and accountability. Rather than uncritically accepting AI developments or unreasonably resisting them, nursing staff should actively participate in the debate and contribute to it, as AI will affect their roles and the nature of their work (Arnold 2021). The involvement of nurses in the development of AI can, therefore, be seen as highly relevant because they possess deep contextual expertise in patient care, workflow integration, and safety‐critical decision‐making. One of the biggest challenges is ensuring that technologies are implemented effectively, equitably, and sustainably in clinical care (Kumar et al. 2023).

Despite its potential, the integration of AI within healthcare settings, particularly among nursing staff, remains minimal. This restrained adoption presents a critical gap in understanding, specifically, how AI influences nurses' critical thinking and decision‐making processes (Secinaro et al. 2021).

The knowledge gap lies in the limited exploration of how AI technologies influence nurses' clinical decision‐making processes and their integration into nursing workflows. Despite the growing interest in AI applications within healthcare, there is a lack of systematic evidence identifying and describing AI technologies specifically designed for nursing practice. This gap hinders the understanding of how these technologies can be effectively utilised to enhance nurses' decision‐making, optimise their workflows, and address the challenges they encounter in clinical settings. This systematic literature review aims to bridge this gap by identifying and describing AI technologies used in nurses' clinical decision‐making within healthcare settings. By synthesising existing evidence, this study seeks to provide a comprehensive understanding of the role AI can play in enhancing nursing practice and informing future research and implementation strategies.

## 2 Methods

This systematic literature review aimed to identify and describe AI technologies for nurses' clinical decision‐making in healthcare settings. The research questions were: (1) what type of AI technology has been developed to support nurses' decision‐making in healthcare settings?; (2) what type of effects have been reached in the decision‐making of nurses while using AI technology in healthcare settings?

The systematic review of interventional studies on the use of AI to support nurses' clinical decision‐making was conducted to provide evidence‐based information on the extent to which AI is being used in healthcare settings and the potential of AI to support future nursing practice. The research data were analysed using appropriate methods to draw unbiased conclusions based on the research evidence (Aromataris and Pearson 2014; Lasserson et al. 2019). The review was conducted according to the Joanna Briggs Institute (JBI 2020) guidelines and reported according to the PRISMA statement (Page et al. 2021).

### 2.1 Search Strategy

Five electronic multidisciplinary databases (CINAHL, PubMed, Scopus, ProQuest and Medic) were searched in May 2024 to identify relevant studies. The PICO format (P = population, I = intervention, C = comparators, O = outcomes) was used to define the research question (see Table 1). Participants were defined as nurses and excluded in case they were other healthcare professionals or students. Interventions were defined by AI solutions or machine/deep learning developed and tested to support nurses' decision‐making in clinical patient care. The outcomes were nurses' critical thinking and/or decision‐making. The type of studies was all kinds of interventional studies, including a randomised controlled trial (RCT) or quasi‐experimental studies. A separate search strategy was developed for each database based on the required keyword vocabulary and MeSH terms according to the PICO inclusion criteria. The search was performed using four keywords and combining them with the Boolean operators AND, OR and NOT (see File S1). For example, the PubMed search included both MeSH terms and text words, such as (“Nurses”[MeSH Terms] OR nursing[Text Word]) AND (“Thinking”[MeSH Terms] OR “critical thinking”[Text Word]) AND (“Computing Methodologies”[MeSH Terms] OR “artificial intelligence”[Text Word] OR “machine learning”[Text Word] OR “deep learning”[Text Word]). Each database‐specific strategy was further refined and adjusted according to its indexing system, search algorithm, and controlled vocabulary requirements. Each search was limited to studies published in either English or Finnish within twenty years of rapid AI development in healthcare, 2005–2024.

**TABLE 1:** Inclusion and exclusion criteria using PICO format.

Criteria	Inclusion	Exclusion
Population	Nurses	Other healthcare professionals
Intervention	Critical thinking and decision‐making with the aid of Artificial intelligence	No AI or machine learning No decision‐making or critical thinking
Comparators	Traditional care without artificial intelligence or no comparison	—
Outcomes	Nurses' critical thinking and/or decision‐making	No nurses' critical thinking or decision‐making
Study design	Randomised controlled trials, Quasi‐experimental studies	Systematic/literature reviews, Qualitative research, Observational, Non‐experimental studies
Publication years	2005–2024	Older
Language	English, Finnish	Other languages

### 2.2 Selection of Studies

**FIGURE 1:**
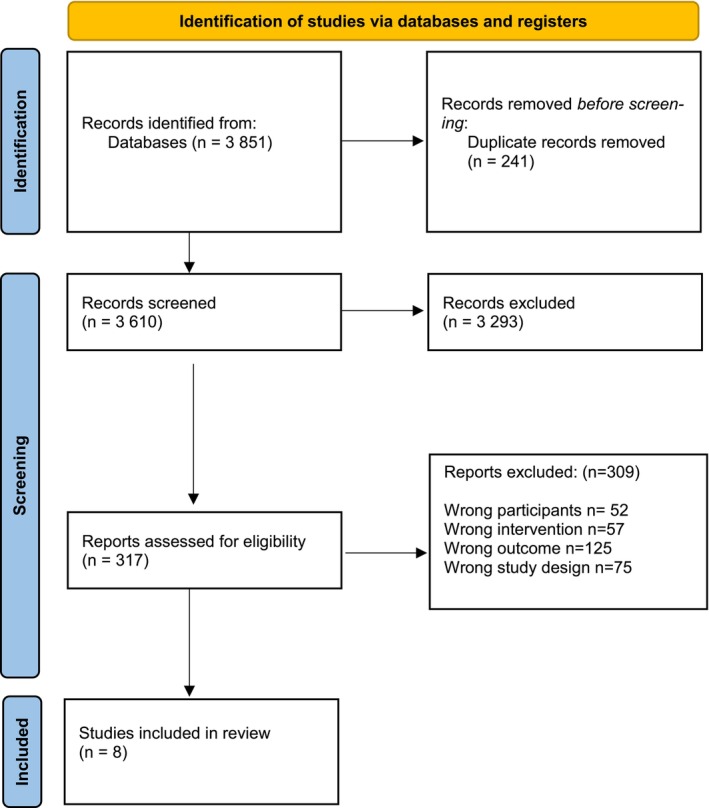
PRISMA flow diagram. [Colour figure can be viewed at wileyonlinelibrary.com]

In the database search, we identified 3851 publications, of which 241 included duplicate records (see PRISMA flow diagram in Figure 1). A total of 3610 studies were screened. At least two researchers independently screened and assessed each publication, meeting the inclusion criteria based on the title and abstract (*n* = 3610) and full text (*n* = 317). A total of eight studies were chosen for narrative synthesis.

### 2.3 Quality Appraisal, Data Extraction and Analysis

The quality appraisal was conducted using JBI Critical Appraisal Checklist for Quasi‐Experimental Studies and/or JBI Critical Appraisal Checklist for RCTs (2017). The JBI checklist for quasi‐experimental studies has nine items covering domains such as temporal precedence, presence of a control group, comparability of participants, outcome measurement, participant follow‐up, and appropriateness of statistical analysis (Barker et al. 2024). The JBI checklist for RCTs has 13 items addressing internal validity (randomisation, allocation concealment, blinding), outcome measurement, participant retention, statistical validity, and trial design (Barker et al. 2023). In the quality appraisal, each checklist item answered ‘yes’ was counted as one point, while responses marked ‘no’ or ‘unclear’ were not scored. The percentage quality score for each study was then calculated by dividing the number of ‘yes’ responses by the total number of items in the respective JBI checklist. The total average of the quality appraisal was 31%. Out of six quasi‐experimental studies, Burns et al. (2022) scored 56%, Auberry and Cullen (2016) and Greenbaum et al. (2019) scored 44%, while the rest of the studies scored 22% or lower (see File S2). RCT studies, Fuerch et al. (2015) scored 23%, and Lopez et al. (2022) scored 0%. Overall, an average quality score of 31% indicates that most studies met only a small proportion of the methodological quality criteria, suggesting a generally low level of evidence reliability. This implies that the available studies provide preliminary but not robust evidence regarding AI effectiveness in nursing decision‐making. The quality appraisal highlighted substantial methodological weaknesses across both quasi‐experimental and RCT designs.

The extraction data table was used to extract the author/s, year of publication, country, the aim of the study, participants, intervention, and key results of the studies selected for the review. The approach to analysing the data was narrative because of the high heterogeneity of the chosen studies and outcomes.

## 3 Results

### 3.1 Characteristics of the Studies

Of the studies included in the final review, six were quasi‐experimental studies (Auberry and Cullen 2016; Bowles et al. 2015; Burns et al. 2022; Greenbaum et al. 2019; Kim et al. 2022; Cho et al. 2015) and the remaining two were RCTs (Fuerch et al. 2015; Lopez et al. 2022). The majority (*n* = 5) were conducted in the USA (Auberry and Cullen 2016; Bowles et al. 2015; Fuerch et al. 2015; Greenbaum et al. 2019; Lopez et al. 2022), while the minority were conducted in Korea (Kim et al. 2022; Cho et al. 2015), and Canada (Burns et al. 2022). This concentration of studies in the United States indicates a geographic bias in the evidence base, suggesting that findings may primarily reflect North American healthcare contexts. The interventions were conducted in healthcare settings; hospitals (Bowles et al. 2015; Burns et al. 2022; Fuerch et al. 2015; Kim et al. 2022; Cho et al. 2015; Lopez et al. 2022), and a trauma centre teaching hospital (Greenbaum et al. 2019). All the included studies were published within the last ten years.

### 3.2 Characteristics of the Participants

Five studies included only registered nurses (Auberry and Cullen 2016; Cho et al. 2015; Greenbaum et al. 2019; Kim et al. 2022; Lopez et al. 2022). Bowles et al. (2015) and Burns et al. (2022) reported the setting of their study and included nursing staff, but they did not specify the exact types of professions. Fuerch et al. (2015) included nurses and other healthcare professionals such as attending physicians, respiratory therapists, consultants, pharmacists, and junior doctors. The number of participants in the included studies ranged from 11 to 222 (total *n* = 1173) in those studies where the numbers of participants were reported as individuals.

### 3.3 Instruments Used to Measure Outcomes

All metrics to measure the results were different (see Table 2), of which a Likert‐type form was used in four studies (Auberry and Cullen 2016; Greenbaum et al. 2019; Kim et al. 2022). Other measures used were the Online Survey software (Lopez et al. 2022), and the Korean version of CAM‐ICU (Cho et al. 2015). Follow‐up was not recorded in any of these studies. This absence of follow‐up data represents a notable gap across the included studies, limiting understanding of the sustained effects of AI interventions on nurses' decision‐making. In addition, data were collected using hospital documents and databases (Bowles et al. 2015) and on paper at the end of each study day (Burns et al. 2022). In one study, two independent trainers videotaped and scored all research situations (Fuerch et al. 2015).

**TABLE 2:** Data extraction.

Author et al. year, country	Aim of the study	Participants	Intervention	Instruments	Outcomes	Pre‐test	Post‐test	Follow‐up	*p*	Key results
Auberry and Cullen 2016, United States	To test the confidence level of nurses prior to implementing the evidence‐based algorithm and three months post implementation of the seizure algorithm	IG: 11 nurses of the Indiana Developmental Disabilities Nurses Association CG: no	Quasi‐experimental, a 3‐month long implementation pilot study of an evidence‐based algorithm for nurses working in the field of intellectual disability	The Surrogate Decision‐Making Self Efficacy Scale (SDM‐SES) is an evidence‐based Likert Scale consisting of five questions with ranked orders from Strongly Agree (4), Agree (3), Disagree (2), to Strongly Disagree (1)	The use of the American Association of Neuroscience Nurses Seizure Assessment Algorithm increases the self‐confidence for many of the nurses in guiding care decisions during telephone trial	The Self‐Efficacy Surrogate Decision Making SelfEfficacy Scale (SDM‐SES)‐ pre‐test: the mean pre‐confidence score 15.6364	The post‐SDM‐SES: the mean post‐confidence score 17.6364	Not reported	The treatment effect was statistically significant −3.169, *p*, 0.01. *p*‐value for the self‐confidence of the nurses not reported	An evidence‐based tool that assists nurses in decision‐making during telephone triage may offer access to appropriate care that is not currently available
Bowles et al. 2015, United States	To develop, instal, and use The Discharge Decision Support System (D2S2) electronically as a software, and to examine it's effect on 30‐ and 60 day readmissions	IG: 6 medical units from three health system hospitals (3 units at the largest facility, 2 units at the second facility, and 1 unit at the third facility). CG: 76 hospital units	Quasi‐experimental, two‐phase study (control and experimental phases) with concurrent comparison units. Control phase 3 weeks (usual care) and experimental phase 4 weeks (intervention units utilised the intervention software while the comparison units continued with usual care)	Referral to traditional post‐acute care and patient refusal were collected by research assistants through chart reviews of the discharge planners´ documentation and from the hospital database. Readmissions were collected from the admission/discharge/transfer data of all subsequent episodes of care, matching on patient name, date of birth, and medical record number. Outcomes were compared for all intervention unit patients before and after the intervention was implemented, and for high‐risk versus low‐risk patients based on Discharge decision support system score	The development, installation and D2S2 use electronically were fully achieved. In 30‐day readmissions, when the D2S2 identified high‐risk patients and the results of screening were provided to the discharge planners, was a statistically significant improvement. The greatest impact was achieved for high‐risk patients. The analysis for 60‐day readmissions showed the same impact	The control phase was studied to establish a baseline about risk status and readmissions. Discharge planners conducted their usual processes with no interview guide or standardised assessment form for this purpose. In the control phase the Discharge Decision Support System (D2S2) scores, answers, and recommendations were not shared with the bedside nurses or discharge planners. Discharge planners conducted their usual processes with no interview guide or standardised assessment form for this purpose. The discharge planners and/or hospitalists made the referral suggestions. The 30‐day readmission rate for intervention unit high‐risk patients: 22.2%	Readmission outcomes compared to before implementation. The 30‐day readmission rate for intervention unit high‐risk patients: 9.4%	Not reported	*p* = 0.015	For clinicians the D2S2 elements were quick and easy to answer, the questions were few, and they were marked with an exclamation mark. Two of the questions were pre‐populated from other places in the electronic health record, leaving only four new items to be documented by the admitting nurses, so the workload was light
Burns et al. 2022, Canada	To develop a technology that would identify illness early, initiate action and therein improve patient care and outcomes, and save healthcare resources	40‐bed medical ward, in a 300‐bed Canadian tertiary care hospital was divided into two groups IG: 163 CG: 110	This was a prospective interventional quality improvement study over a one‐year period. The intervention was an algorithm and software programme capable of detecting the sentinel change in a deteriorating patient's clinical condition and, once detected, directing early investigation and care	Data were manually entered into the program. If a patient is stable, the software program produces a green message. When the software detects a mild to moderate abnormality, it produces a yellow message, directing the user to order level 1 investigations. When the software detects a moderate to severe abnormality, it produces a red message directing the user to order level 1 and level 2 investigations and to contact senior medical staff urgently. All patient data points were recorded on a paper worksheet that was gathered and collated at the end of every day by a research coordinator	A novel algorithm and software used by nursing staff identified acute illness with adequate sensitivity and specificity to reduce ICU transfers and time to clinical intervention on a medical ward	Not reported	Not reported	There was no cross‐over or loss to follow‐up	The time required to order investigations *p* = 0.049, contact senior medical staff *p* = 0.040 and senior medical staff intervention *p* = 0.045	An intelligent, action‐based algorithm, combined with an easy‐to‐use software interface, can increase the detection of acute illness, reduce critical care admissions, and thus reduce patient morbidity and mortality
Cho et al. 2015, Korea	To assess the effects of the implementation of the Automatic Prediction of Delirium in Intensive Care Units (APREDEL‐ICU) system on nursing sensitivity and patient outcomes in surgical intensive care units (SICU). Also evaluating the systems usability and nurse satisfaction with it	Before implementation: 724 patients After implementation: 1111 patients 42 nurses were participating in the system evaluation survey	Quasi‐experimental, pre‐post research design was used in this study to evaluate the clinical impact of the APREDEL‐ICU system by comparing individuals in the research units before and after the introduction of the system. The research period before the installation of the system from January to December in 2010, and after installation of the system from May 2011 to April 2012	The Korean version of the CAM‐ICU was used to measure the occurrence of delirium. Each patients data were extracted from the electronic medical record. In this study was used a pre‐post research design to evaluate the clinical impact of the APREDEL‐ICU system by comparing individuals in the SICU before and after the introduction of the system. Nurses' evaluation of the APREDEL‐ICU system were measured by questions regarding the ease of the use of the system and their overall satisfaction with the system. Selfassessment were also conducted to assess changes in knowledge regarding delirium and opinions concerning the system with a Likert scale, with points ranging from 1 to 5 (i.e., strongly disagree to strongly agree). A higher score indicated greater nurse satisfaction with the system	After the implementation of the Automatic Prediction of Delirium in Intensive Care Units, high‐risk patients, determined using a prediction algorithm, showed a slight decrease in the incidence of delirium, but the changes were not significant. After the implementation significant decreases in the number and duration of analgesic/narcotic therapies were observed. Nurse self‐evaluation results showed an improvement in all categories of knowledge regarding delirium care		Nurse self‐evaluation results showed an improvement across all surveyed categories. Nurses' responses with experience and evaluation of the APREDEL‐ICU system indicated that the system heightened their interest in delirium and encouraged them to pay greater attention to monitoring of the patients	No		The use of a delirium prediction and alerting system brought up the potential impact of increasing the quality of delirium care by the implementation of early detection and proper intervention. Nurses' self‐evaluation results showed an improvement across all surveyed categories, including delirium risk factors and prevention, nursing intervention for delirious patients, delirium prediction and delirium discrimination. The responses indicated that the system heightened nurses' interest in delirium and encouraged them to pay more attention to monitoring of the patients. Also the nurses felt that although they wanted to use the system, it substantially increased their workload
Fuerch et al. 2015, United States	To evaluate adherence to the Neonatal Resuscitation Program algorithm by participants working from memory as compared to participants using a decision support tool that provides visual and auditory guides during simulated neonatal resuscitation	A total of 65 participants: 18 residents, 1 fellow, 7 attending physicians, 2 respiratory therapists, and 37 nurses, from three different neonatal intensive care units. IG:35 CG:30	Randomised controlled design Healthcare professionals with a current neonatal resuscitation programme (NRP) card were randomised to the control or intervention group and participated in three simulated neonatal resuscitation scenarios (A, B, C) in random order. All participants in both control and intervention groups were presented with the same three scenarios. The vital signs were pre‐determined and preprogrammed into the NeoCue device and didn't vary	All scenarios were video recorded, and the recordings were independently reviewed and scored by two NRP instructors. A score was assigned for both positive pressure ventilation (PPV) and chest compressions (CC) for each subject based on the number of seconds of appropriate activity during each scenario. The highest possible score was 270, based upon the number of seconds in a 4.5 min scenario. For FiO2 adjustment, each subject received a score based on whether the FiO2 was addressed at least once during each minute interval of the 4‐min scenario. All of these scores were compared between the two groups by Wilcoxon rank sum (Mann–Whitney) test. For ease of interpretation, the PPV and CC scores were converted to a percentage for presentation. Stata/SE 12.0 (College Station, TX) was used for statistical analysis	Participants using a decision support tool exhibit significantly fewer deviations from the NRP algorithm compared to those working from memory alone during simulated neonatal resuscitation	No pre‐test	No post‐test	Not reported	Positive pressure ventilation was performed correctly 55%–80% of the time in the control group vs. 94%–95% in the intervention group across all three scenarios (*p* < 0.0001). Chest compressions were performed correctly 71%–81% of the time in the control group vs. 82%–93% in the intervention group in the two scenarios in which they were indicated (*p* < 0.0001). FiO2 was addressed three times more frequently in the intervention group compared to the control group (*p* < 0.001)	Using a decision support tool during actual neonatal resuscitation may improve human performance in clinical practice by reducing errors
Greenbaum et al. 2019, United States	To determine the effect of domain‐specific ontology and machine learning‐driven user interfaces on the efficiency and quality of documentation of presenting problems in the emergency department (ED)	The study was performed in a 55,000 visits/year Level I trauma center and tertiary, academic, adults‐only, teaching hospital. The participants were nurses	Quasi‐experimental, a mixed methods study that consisted of both a retrospective cohort before‐and‐after design and a qualitative study. The study was comprised of a (1) pre‐implementation period 12 months (2) a development period 32 months (3) and a post‐implementation period. The study compares outcomes measures between the pre‐implementation and post‐implementation periods (16 month period following the completion of the final version of the intervention)	Patient level outcomes were measured as positive if all of the documented presenting problems listed for the patient were able to be mapped to the ontology. If any of the presenting problems were not mapped, was the outcome considered to be negative. For the qualitative component, every reviewer independently assessed every chart's presenting problem (completeness, precision, and overall quality). Each of these metrics was scored on a four‐point likert scale	A domain‐specific ontology and machine‐learning‐driven user interface improved structured data capture, ontology usage compliance, and data quality	Completeness 3.35 Precision 3.59 Overall quality 3.38	Off/on Completeness 3.54/3.66 Precision 3.67/3.74 Overall quality 3.58/3.72	Not reported	After deployment, while the system was operational, performance improvement was 97.2%, *p* < 0.0001	Machine learning‐driven user interfaces can be effective for presenting problems and can almost certainly be expanded to additional areas such as diagnosis, procedures, and problem lists to streamline user data entry and improve ontology adherence
Kim et al. 2022, Korea	To develop and evaluate the effectiveness of a clinical decision support system for pressure ulcer prevention on clinical workflow	49 registered nurses, working in seven tertiary hospitals and five secondary hospitals. IG: 23 CG: 27	A quasi‐experimental study, with a non‐equivalent control group, non‐synchronised design. Snowball sampling was used to recruit the study participants. Data was collected between January and April 2020	The format and content were evaluated using eight items by three experts after a short period of implementation of the clinical decision support system. The format (overall impression, download and switch speed, accessibility, and convenience) and the contents usefulness (clarity, applicability) and reliability (up‐to‐date, accurate) were evaluated by using a 5‐point Likert scale. For decision‐making a 24‐item decision‐making instrument was used (developed by Lauri and Salanterä). Study used a 5‐point Likert Scale. Scores for responses to odd items were reserved, and those for even items were not changed. A high score: an intuitive approach to decision‐making, low score: an analytic approach	The level of pressure ulcer prevention nursing performance and visual differentiation ability of skin pressure and oral mucosa pressure ulcer showed significantly greater improvement in the experimental group compared with the control group, whereas clinical decision making did not differ significantly. A clinical decision support system using machine learning was partially successful in performance of skin pressure ulcer prevention, attitude, and visual differentiation ability for skin and oral mucosa pressure ulcer prevention	Baseline measures of demographics and outcome variables for all participants and a comparison of scores for the two groups were performed. There were no other significant differences between the two groups, than the mean age (*t* = 5.57, *p* < 0.001)	Knowledge of PU prevention practice (*t* = −0.09, *p* = 0.928) Attitude towards oral mucosa PU prevention (*t* = 0.65, *p* = 0.519) Attitude towards PU prevention was better for the experimental group than the control group (*t* = −7.37, *p* < 0.001). The degree of PU prevention nursing performance (*t* = −3.30, *p* = 0.002) and the visual differentiation ability of skin PUs (*t* = −4.01, *p* < 0.001) and of oral mucosa PUs (*z* = −2.02, *p* = 0.044) clinical decision‐making (*t* = −1.01, *p* = 0.316)	Not reported	Knowledge of PU prevention practice (*t* = −0.09, *p* = 0.928) Attitude towards oral mucosa PU prevention (*t* = 0.65, *p* = 0.519) Attitude towards PU prevention was better for the experimental group than the control group (*t* = −7.37, *p* < 0.001). The degree of PU prevention nursing performance (*t* = −3.30, *p* = 0.002) and the visual differentiation ability of skin PUs (*t* = −4.01, *p* < 0.001) and of oral mucosa PUs (*z* = −2.02, *p* = 0.044) clinical decision‐making (*t* = −1.01, *p* = 0.316)	Study findings suggest that a CDSS using machine learning needs to be implemented for skin and oral mucosa PU prevention in clinical settings
Lopez et al. 2022, United States	To compare effectiveness of presenting clinical evidence in four formats (text, text + table, text + graph, and tailored)	IG: 220 hospital based registerd nurses (recruit using state based professional registries, participants from multiple institutions across the nation) CG: no	A randomised controlled trial (RCT) design, registered nurses were randomised into one of four groups. All participants in the clinical decision support (CDS) groups 1–3 thus access CDS evidence presented in the format associated with that group. Group 4 were presented with one the three CDS formats based on their Graph Literacy Scale scores (low, medium, or high). The GLS is a 13‐item objective literacy tool that measures a person's ability to understand information presented in graphs	Online survey software (REDCap) was used for efficient study workflow including screening, documentation and data collection. The CDS prototype was accessed via a web app and the simulation‐based experiment was conducted remotely at a subject's local computer using video‐conferencing software. The CDS prototype was accessed via a web app and the simulation‐based experiment was conducted remotely at a subject's localcomputer using video‐conferencing software	Results?	The informed consent, GLS, and the additional demographic questions	6 post intervention surveys for assessment of cognitive workload usability, numeracy, format preference, CDS utilisation rationale, and CDS interpretation	Not reported		

### 3.4 Presentation of Results

Various studies have explored technology implementation in nursing decision‐making, each contributing unique insights into its effects. These studies can be categorised into (1) *specialised* versus *generalised decision support tools*, (2) *predictive technologies, and* (3) *usability and workflow efficiency enhancements*. A critical comparison of these studies reveals the strengths and limitations of different technological interventions and their practical implications in nursing.

#### 3.4.1 Specialised Versus Generalised Decision Support Tools

The studies by Bowles et al. (2015) and Burns et al. (2022) illustrate the contrast between specialised and generalised AI tools in improving diagnostic outcomes. Bowles et al.'s Discharge Decision Support System (D2S2) is a specialised tool that significantly improved 30‐ and 60‐day readmission rates by focusing on post‐acute service referrals. In contrast, Burns et al. developed a broader software program for identifying acute illness, which successfully reduced ICU transfers and time to clinical intervention. The broader applicability of Burns et al.'s software allows it to be used in various clinical settings, whereas the specialised D2S2 shows a higher impact on specific outcomes. This comparison highlights the trade‐off between the depth of impact of specialised tools versus the versatility of generalised tools. Lopez et al. (2022) developed a tailored AI‐driven clinical decision support (CDS) system for nurses by leveraging data from a national RCT to ensure relevance and accuracy in diverse healthcare settings. The CDS system was designed to provide real‐time, evidence‐based recommendations, supporting nurses in making informed decisions remotely. By conducting the study on a national scale, the researchers demonstrated the feasibility and implications of implementing AI solutions to enhance clinical decision‐making in nursing practice.

#### 3.4.2 Predictive Technologies

Kim et al. (2022) and Cho et al. (2015) explored advanced predictive technologies with differing results. Kim et al.'s machine learning‐based CDSS for skin pressure ulcer prevention demonstrated partial success, improving nursing performance and attitudes but facing challenges in clinical integration. Conversely, Cho et al.'s APREDEL‐ICU, a traditional predictive algorithm, significantly reduced analgesic treatments and increased nurses' knowledge of delirium. This suggests that while machine learning holds potential, simpler predictive models might offer more consistent and practical benefits, emphasising the need for more straightforward solutions that can be seamlessly integrated into clinical practice.

#### 3.4.3 Usability and Workflow Efficiency Enhancements

Fuerch et al. (2015) emphasised the importance of user interface and training. Fuerch et al.'s decision‐support tool for neonatal resuscitation reduced protocol deviations and improved adherence to clinical guidelines. Greenbaum et al. (2019) reinforced this by demonstrating that tailored interfaces significantly enhance data collection and quality, particularly in high‐pressure environments like emergency departments. Auberry and Cullen (2016) highlight how transparent and evidence‐based decision‐support tools boost nurse confidence and minimise errors. Auberry and Cullen's seizure algorithm increased confidence in treatment decisions. These findings collectively underscore the direct link between tool clarity and nurse performance.

### 3.5 Effects of Interventions

Across the eight studies, the interventions demonstrated significant improvements in clinical decision‐making, patient care, and staff performance outcomes. For instance, Bowles et al. (2015) implemented the Discharge Decision Support System (D2S2), which reduced 30‐day readmission rates for high‐risk patients from 22.2% to 9.4%, achieving a statistically significant *p*‐value of 0.015. Similarly, Burns et al. (2022) developed an algorithm to detect patient deterioration, significantly reducing the time to contact senior medical staff (*p* = 0.040) and to order investigations (*p* = 0.049). In another example, Fuerch et al. (2015) evaluated a neonatal resuscitation decision support tool, finding improved adherence to resuscitation protocols; positive pressure ventilation was correctly performed 94%–95% of the time in the intervention group compared to 55%–80% in the control group (*p* < 0.001).

Auberry and Cullen (2016) reported a significant increase in nurses' confidence using an evidence‐based seizure assessment algorithm, with SDM‐SES confidence scores improving from 15.6364 to 17.6364 (*p* = 0.01). Cho et al. (2015) highlighted enhanced knowledge and attention to delirium care following the implementation of the APREDEL‐ICU system, though no significant reductions in delirium incidence were observed. Kim et al. (2022) demonstrated significant improvements in nursing performance for pressure ulcer prevention (*p* = 0.002) and visual differentiation of skin ulcers (*p* < 0.001) using a machine‐learning‐based clinical decision support system, though clinical decision‐making scores showed no significant change (*p* = 0.316).

Greenbaum et al. (2019) focused on documentation efficiency, showing that a domain‐specific ontology and machine‐learning‐driven interface significantly improved documentation quality, with metrics such as precision increasing from 3.59 to 3.74 (*p* < 0.001). Lopez et al. (2022) emphasised the importance of tailored presentation formats in clinical decision support systems, improving usability and decision‐making efficiency, although specific *p*‐values were not reported.

Collectively, these studies demonstrate the potential of decision support systems to enhance clinical decision‐making, improve patient care, and optimise workflows. They also underscore the importance of tailoring interventions to user needs while addressing challenges such as usability and workload integration to achieve sustained success.

## 4 Discussion

The systematic review addressed a significant knowledge gap in understanding the role and impact of AI technologies in nursing clinical decision‐making. Despite the increasing interest in AI applications within healthcare, there is limited systematic evidence identifying and describing AI technologies specifically tailored to nursing practice. This gap has hindered efforts to effectively integrate these technologies into nursing workflows, optimise decision‐making processes, and address challenges faced by nurses in clinical settings. By conducting a narrative synthesis of the findings, the review categorised the results into three main types of interventions used for AI technology: (1) specialised versus generalised decision support tools, (2) predictive technologies, and (3) usability and workflow efficiency enhancements. The predominance of studies from the United States also highlights the need for broader, cross‐cultural validation of these findings to ensure transferability across diverse healthcare systems.

Technologies that affect nurses' decision‐making are an increasing trend worldwide. However, beyond acknowledging AI's potential, its integration into nursing practice requires structural and educational readiness. Incorporating AI into nursing requires many steps, including curricular reform within academic institutions and clinical practice settings. This reform will ensure that nursing students and practising nurses can work safely and efficiently in the age of AI. Nurse educators should adopt new and evolving pedagogies that leverage AI's unique features (Buchanan et al. 2020; Ronquillo et al. 2021). From the perspective of healthcare organisations, their role is to harness AI's power to deliver maximum value for caretakers and patients (McGrow 2019). Concrete policy initiatives, such as integrating digital literacy standards into national nursing curricula or aligning organisational AI strategies with WHO's 2023 guidance on ethics and governance of AI in health, could accelerate readiness at both system and institutional levels.

This systematic review evaluated the effects of AI on nurses' decision‐making in healthcare environments, based on eight studies published between 2015 and 2022. The results demonstrate that AI can significantly support nurses in areas such as diagnostic outcomes, patient care, and workflow efficiency. However, there was wide variation in study designs, research questions, intervention durations, and outcome measures, and notably, none of the studies included follow‐up assessments, limiting understanding of long‐term effects. This underscores the need for standardised, high‐quality studies to validate AI applications in future research.

The studies revealed distinct differences between specialised and generalised decision support tools. For example, Bowles et al. (2015) demonstrated the effectiveness of the specialised Discharge Decision Support System, which significantly reduced 30‐day readmission rates for high‐risk patients from 22.2% to 9.4%. In contrast, Burns et al. (2022) developed a generalised algorithm to detect patient deterioration, which reduced ICU transfers and shortened the time to initiate clinical interventions significantly. These findings highlight the trade‐offs between the depth of impact on specialised tools and the broader applicability of generalised solutions.

Usability and integration into clinical workflows emerged as key factors influencing the success of AI interventions. Tools with user‐friendly interfaces and tailored designs, such as the neonatal resuscitation decision support tool by Fuerch et al. (2015), achieved significant improvements in adherence to clinical guidelines. Similarly, Auberry and Cullen (2016) found that an evidence‐based seizure assessment algorithm significantly boosted nurse confidence. These findings suggest that user interface design and seamless workflow integration are critical determinants of success. Tools that are intuitive, require minimal additional documentation, and align with established nursing processes are more likely to be adopted and sustained. Conversely, poorly integrated or complex systems risk rejection, regardless of their technical sophistication. However, Cho et al. (2015) reported increased workload associated with their delirium prediction tool, despite improvements in nurses' knowledge and attention to delirium care. These findings emphasise the need for AI tools that not only enhance performance but also minimise disruptions to workflow. This further underlines that effective AI deployment depends as much on organisational readiness and leadership commitment as on technical accuracy.

Ethical considerations extend beyond technical reliability to questions of accountability, fairness, and trustworthiness. Ensuring the ethical and moral safety of AI systems is paramount, with the reliability and validity of the datasets being crucial for their development and application. Transparency and data sharing can contribute to safety, ideally achieved through open access to both data and algorithms. However, justifiable reasons, such as the protection of intellectual property and the need to mitigate cybersecurity risks, may necessitate certain restrictions. Beyond transparency, issues such as algorithmic bias, explainability, and equitable access require systematic attention. For example, national frameworks such as the European Union's AI Act (European Parliament and Council of the European Union 2024) or Finland's ethical AI principles (Ministry of Economic Affairs and Employment of Finland 2021) provide models for regulating algorithmic accountability and bias mitigation. In nursing contexts, clear protocols defining professional responsibility for AI‐supported decisions would help safeguard patient safety and clarify liability. Third‐party or governmental audits could provide a balanced approach to addressing these concerns. Furthermore, transparency regarding the types of data used in AI development, as well as the limitations of the software, remains essential (Gerke et al. 2020). Embedding ethical review processes within institutional governance, alongside technical evaluation, can ensure that AI adoption aligns with professional standards and human rights principles.

Digital technology knowledge and skills have the potential to significantly improve patient care, but healthcare professionals must recognise the benefits of using such technologies to foster positive attitudes and motivation towards their integration into practice. Organisations play a critical role in this process by providing adequate resources, equipment, facilities, and time to support the adoption and effective use of technologies (Konttila et al. 2019). Instruments such as DigiHealthCom and DigiComInf have been developed to measure healthcare professionals' digital health competence and associated educational and organisational factors (Jarva et al. 2023). Measuring healthcare professionals' self‐assessed competence in digitalisation is increasingly necessary as healthcare practices become more digitalised. Digital health competence is gradually being recognised as a core competence for healthcare professionals, and these instruments could also be utilised to assess and enhance the digital health skills required to meet future needs.

Future research should focus on developing rigorous, longitudinal, and cross‐national studies to assess both the short‐ and long‐term effects of AI tools on nursing decision‐making and patient outcomes. The active involvement of nurses in the development of AI solutions is crucial to ensuring that these systems address patient care needs effectively and align with clinical workflows. Involvement should go beyond consultation to co‐design, where nurses collaborate in model validation, ethical evaluation, and implementation planning. Additionally, strategies for engaging nurses across diverse job roles to safely learn and adopt new systems are essential for successful implementation. Concrete mechanisms could include national competency frameworks, pilot projects in clinical education, and policy incentives supporting AI training. Exciting areas for AI research include addressing ethical questions and safety concerns. For instance, if a study is conducted among a limited population, there is a risk that the AI system could produce biased assumptions, potentially leading to treatment errors. Moreover, with patients becoming more aware of their rights, concerns about data processing, privacy, and security are growing. These issues must be carefully considered in the design and deployment of AI‐based decision support systems. Developing explainable and auditable AI systems that support professional accountability and patient autonomy will be critical for maintaining public trust.

### 4.1 Limitations and Strength

This study has several limitations. The search was restricted to articles in English and Finnish, and all selected articles were in English. Additionally, the identified studies were conducted in only four countries, limiting the global generalisability of the findings. Many of the included studies failed to meet quality appraisal scores, often lacking control groups, pre‐ and post‐measurements, or sufficient reporting of participant follow‐up. The included RCTs scored poorly due to inadequate randomisation procedures, lack of blinding, incomplete follow‐up, and limited transparency in reporting. The quality assessment outcomes indicate that the body of evidence is at high risk of bias, which diminishes confidence in the reported effectiveness of AI interventions to support nurses' decision‐making. This underscores the urgent need for future studies to apply more rigorous designs and to report methodological details transparently in order to strengthen the evidence base. Additionally, the heterogeneity in research questions, outcomes, and measurement methods among the studies made it challenging to compare results and precluded the feasibility of a meta‐analysis.

Important to acknowledge that the wide range of measurement tools used across studies poses challenges for reliability and comparability. While some tools, such as the CAM‐ICU, are standardised and validated, others (e.g., study‐specific Likert‐type scales or ad hoc online surveys) lack established psychometric properties. This heterogeneity reduces confidence in the consistency of findings and complicates synthesis across studies, thereby limiting the strength of conclusions that can be drawn.

A critical limitation across the included studies was the lack of systematic follow‐up. Only four out of eight studies reported any follow‐up data, and even then, the periods were short and inconsistent. This absence prevents evaluation of whether the effects of AI interventions on nurses' decision‐making were sustained over time or whether they diminished once the intervention ended. Without longitudinal evidence, it is difficult to determine the durability, transferability, and real‐world impact of these tools in clinical practice. Future studies should therefore incorporate longer‐term follow‐up assessments with standardised time points to capture both immediate and sustained outcomes.

The geographic distribution of included studies represents a critical limitation for the global applicability of our findings. With the majority of studies conducted in the United States and only a few from Korea and Canada, the evidence is heavily weighted towards North American healthcare contexts. This concentration raises concerns about the transferability of results to other regions, where differences in healthcare systems, nursing roles, digital readiness, and cultural attitudes towards technology may influence the effectiveness and uptake of AI‐based decision support. Therefore, while the findings provide valuable preliminary insights, they should be interpreted with caution when considering global implementation, and further research is needed across diverse international settings.

### 4.2 Future Directions

1. Future research should prioritise the involvement of nurses in the development and refinement of AI tools. Engaging end‐users in the design process can ensure that these systems are tailored to clinical needs, enhancing their relevance, usability, and adoption. Studies like Greenbaum et al. (2019) have demonstrated the effectiveness of tailored interfaces in improving documentation quality (*p* < 0.001), underscoring the importance of aligning technology with the practical workflows of nursing professionals.
2. The ethical dimensions of AI in healthcare require significant attention. Research should focus on addressing data security, privacy, and potential biases in AI algorithms. Limited study populations, as highlighted in Auberry and Cullen (2016), risk creating algorithms that generate inaccurate or harmful assumptions, leading to errors in patient care. Ensuring robust ethical frameworks and rigorous testing is essential to safeguard patient outcomes and maintain trust in AI systems.
3. Building on the work of Jarva et al. (2023), integrating digital health competence into nursing education and professional development is essential. Digital skills should be treated as core competencies for nurses, equipping them to effectively leverage AI technologies in their practice. This integration will ensure that nurses are prepared to navigate and utilise increasingly digitalised healthcare environments safely and efficiently.

## 5 Conclusion

The use of AI to support nurses' decision‐making should be significantly strengthened in the future, given that nurses represent the largest professional group in healthcare. From the nurses' perspective, integration of AI into practice must be designed to reduce workload, enhance the meaningfulness of work, and improve retention rates. This could also help address ongoing resource challenges in healthcare by improving efficiency and job satisfaction.

Successful implementation, however, depends on producing higher‐quality evidence that reflects nurses' perspectives and clinical realities. Future research should employ robust methodologies, such as adequately powered randomised controlled trials, mixed‐methods designs, and longitudinal follow‐up, to examine both the effectiveness and sustainability of AI tools. Actively involving nurses in co‐design and usability testing remains essential to ensure that technologies are acceptable, intuitive, and aligned with real‐world workflows.

At the practice level, healthcare organisations must invest in continuous digital competence training and create opportunities for nurses to participate in AI‐related innovation projects. For policymakers, the next step is to translate ethical principles into clear governance mechanisms that define accountability, transparency, and equitable access, building on regulatory frameworks such as the EU AI Act and national digital strategies. Within education, nursing curricula should integrate data literacy and AI ethics to prepare future professionals for increasingly digital care environments, while researchers should expand interdisciplinary collaborations to evaluate the long‐term clinical and ethical impacts of AI.

Collective action across these domains is crucial. By combining policy leadership, organisational commitment, and professional engagement, AI can evolve from a technological promise into a trusted instrument of evidence‐based, ethical, and compassionate nursing care. This transformation will require persistence, collaboration, and a shared vision of technology that amplifies, rather than replaces, the human judgement and empathy at the core of nursing practice.

## Funding

This work was supported by Finnish National Agency for Education (Grant OPH‐270‐2020) and The University of Oulu & The Research Council of Finland Profi 7 (352788).

## Conflicts of Interest

The authors declare no conflicts of interest.

## Supporting information


**Data S1:** jocn70156‐sup‐0001‐Supinfo01.docx.

**Data S2:** jocn70156‐sup‐0002‐Supinfo02.docx.

## Data Availability

Data sharing not applicable to this article as no datasets were generated or analysed during the current study.
