# Supplementary material for: Artificial Intelligence Technologies Supporting Nurses' Clinical Decision‐Making: A Systematic Review
Source: J Clin Nurs. 2025 Nov 25;35(4):1525–40. doi: 10.1111/jocn.70156 (PMC12964510; doi:10.1111/jocn.70156)
Supplement: Supplementary file 2 — Data S2: jocn70156‐sup‐0002‐Supinfo02.docx. [file JOCN-35-1525-s002.docx]

Supplementary File 1

Search strategy used in the electronic databases

Search strategy

*CINAHL* (MH "Nurses+") OR (nursing OR nurses OR nurs* OR practical nurs* OR registered nurs* OR licensed nurs* OR nurs* practitioner OR public health nurs* OR occupational health care nurs*) AND (MH "Thinking+") OR (critical thinking OR decision making OR decision support* OR decision tree OR decision diagram) AND (MH "Computing Methodologies+") OR (artificial intelligence OR ai OR a.i. OR machine learning OR deep learning)

*PubMed* (“Nurses” [MeSH Terms]) OR (nursing [Text Word ]) OR (nurses [Text Word ]) OR (nurs*[Text Word]) OR (practical nurs*[Text Word ]) OR (registered nurs*[Text Word ]) OR (licensed nurs*[Text Word ]) OR (nurs* practitioner[Text Word ]) OR (public health nurs*[Text Word ]) OR (occupational health care nurs*[Text Word ]) AND (“Thinking” [MeSH Terms]) OR (critical thinking[Text Word ]) OR (decision making[Text Word ]) OR (decision support*[Text Word ]) OR (decision tree[Text Word ]) OR (decision diagram[Text Word ]) AND ("Computing Methodologies"[MeSH Terms]) OR (artificial intelligence [Text Word ]) OR (ai[Text Word ]) OR (a.i. [Text Word ]) OR (machine learning[Text Word ]) OR (deep learning[Text Word ])

*Scopus* TITLE-ABS-KEY (nursing OR nurses OR nurs* OR “practical nurs*” OR “registered nurs*” OR “licensed nurs*” OR “nurs* practitioner” OR “public health nurs*” OR “occupational health care nurs*”) AND TITLE-ABS-KEY (Thinking OR “critical thinking” OR “decision making” OR “decision support*” OR “decision tree” OR “decision diagram”) AND TITLE-ABS-KEY (“Computing Methodologies” OR “artificial intelligence” OR ai OR “machine learning” OR “deep learning”)

*Medic* hoitotyö* AND (kriittinen ajattelu OR päätöksenteko) AND (laskennallinen ajattelu OR tekoäly)

*ProQuest* (nursing OR nurses OR nurs* OR “practical nurs*” OR “registered nurs*” OR “licensed nurs*” OR “nurs* practitioner” OR “public health nurs*” OR “occupational health care nurs*”) AND (Thinking OR “critical thinking” OR “decision making” OR “decision support*” OR “decision tree” OR “decision diagram”) AND (“Computing Methodologies” OR “artificial intelligence” OR ai OR a.i. OR “machine learning” OR “deep learning”)

Supplementary File 2

Assessment of methodological quality of the included studies

| **JBI Critical Appraisal Checklist for Quasi-Experimental Studies** | Auberry & Cullen 2016 | Bowles et al. 2015 | Burns et al., 2022 | Cho et. al. 2014 | Greenbaum et al. 2019 | Kim et al. 2022 |
| --- | --- | --- | --- | --- | --- | --- |
| 1. Is it clear what is the ‘cause’ and what is the ‘effect’? | low | low | low | low | high | low |
| 2. Were the participants included in any comparisons similar? | low | low | low | low | low | low |
| 3. Were the participants included in any comparisons receiving similar treatment, other than the intervention of interest? | low | high | unclear | high | high | low |
| 4. Was there a control group? | high | low | low | high | high | low |
| 5. Were there multiple measurements of the outcome both pre and post the intervention? | high | low | low | low | high | low |
| 6. Was follow up complete and if not, were differences between groups in terms of their follow up adequately described and analyzed? | high | high | high | low | low | unclear |
| 7. Were the outcomes of participants included in any comparisons measured in the same way? | low | low | high | low | low | low |
| 8. Were outcomes measured in a reliable way? | low | low | high | low | low | low |
| 9. Was appropriate statistical analysis used? | high | low | high | low | low | low |
| % | 44 | 22 | 56 | 22 | 44 | 0 |

| **JBI Critical Appraisal Checklist for RCT Studies** | Fuerch et al. 2015 | Lopez et al. 2022 |
| --- | --- | --- |
| 1. Was true randomization used for assignment of participants to treatment groups? | unclear | low |
| 2. Was allocation to treatment groups concealed? | high | low |
| 3. Were treatment groups similar at baseline? | unclear | unclear |
| 4. Were participants blind to treatment assignment? | high | unclear |
| 5. Were those delivering treatment blind to treatment assignment? | high | unclear |
| 6. Were outcomes assessors blind to treatment assignment? | low | unclear |
| 7. Were treatment groups treated identically other than the intervention of interest? | low | low |
| 8. Was follow up complete and if not, were differences between groups in terms of their follow up adequately described and analyzed? | low | unclear |
| 9. Were participants analyzed in the groups to which they were randomized? | low | unclear |
| 10. Were outcomes measured in the same way for treatment groups? | low | low |
| 11. Were outcomes measured in a reliable way? | low | low |
| 12. Was appropriate statistical analysis used? | low | low |
| 13. Was the trial design appropriate, and any deviations from the standard RCT design accounted for in the conduct and analysis of the trial? | low | low |
| % | 23 | 0 |
